# Comparison Between Creatinine-Modified Pugh Score and Child-Pugh Score for Prognostication in Decompensated Cirrhosis

**DOI:** 10.7759/cureus.62311

**Published:** 2024-06-13

**Authors:** Suraj Kumar, Shobhit Shah, Balvir Singh, Akshyaya Pradhan

**Affiliations:** 1 Cardiology, King George's Medical University, Lucknow, IND; 2 Medicine, Autonomous State Medical College, Firozabad, IND

**Keywords:** blood creatinine level, hepatic encephalopathy, child-pugh, chronic liver disease, cirrhosis

## Abstract

Introduction, aim, and objective: Despite recent evidence suggesting the blood creatinine level is a significant predictor of survival in liver cirrhosis patients, the conventional Child-Pugh (CP) score has held a longstanding position as a valuable prognostic indicator in cirrhotic individuals. This study aimed to compare the predictive capabilities of the modified CP score and the traditional CP score in decompensated cirrhosis patients to evaluate their prognostic power. The objective of this study was to assess the prognostic value of the modified and traditional CP scores in individuals with decompensated cirrhosis by assessing their predictive accuracy.

Methods: A total of 100 patients diagnosed with decompensated cirrhosis participated in this prospective study. Each patient's Child-Pugh score and class were determined using admission data, with scores ranging from 5 to 15. Serum creatinine was incorporated as the sixth variable to compute the modified CP score, which ranges from 5 to 19.

Results: The percentages of individuals aged 16-30, 31-40, 41-50, 51-60, and above 60 years were as follows: 16.0%, 29.0%, 26.0%, and 11.0%, respectively. The patients had a mean age of 44.71 years and a standard deviation of 13.40 years. Out of the 100 patients studied, 26% were female and 74% were male. Fifty-two percent of patients had mild hepatic encephalopathy, while 24% had moderate encephalopathy and 24% had severe encephalopathy. In cases of moderate and severe hepatic encephalopathy, the creatinine-modified Pugh score showed a considerably large area under the curve (AUC=0.852) on the receiver operating characteristics (ROC) curve.

Conclusion: When blood creatinine is taken into account, it can enhance the Child-Pugh classification's prognostic usefulness. This is especially true for patients with moderate to severe hepatic encephalopathy, where serum creatinine is a key factor in accurately predicting both survival and complications associated with cirrhosis.

## Introduction

Cirrhosis and chronic liver disease (CLD) account for roughly two million fatalities yearly on a global scale [1,2]. Most of the surge in CLD mortality has been seen in nations located in Asia and Africa [3]. In Asian Nations like India, alcohol ranks as the most prevalent cause of chronic liver illness [4]. Patients with compensated cirrhosis have a significantly longer median survival of nine to 12 years, in contrast to individuals with decompensated cirrhosis, who have an average survival of only two years. The clinical stage of the disease and the presence of comorbidities influence the prognosis. The Child-Pugh (CP) classification has replaced the Child-Turcotte classification, which was widely used to evaluate disease severity in patients with end-stage liver disease from 1973 onwards. The CP classification incorporates prothrombin time (PT) as an indicator of nutritional status, replacing the original nutritional status factor [5].

The modified Child-Pugh (CP) score and the Child-Turcotte classification are widely used in everyday clinical practice due to their practicality. These are the most frequently employed prognostic indicators for patients with decompensated cirrhosis, as evidenced by studies [6-8]. The CP score, a comprehensive measure, takes into account serum bilirubin levels, albumin levels, prothrombin time prolongation, hepatic encephalopathy, and ascites severity. It ranges from 5 to 15. Patients are classified into three groups based on their CP score as follows: Class A (5-6), Class B (7-9), and Class C (10-15). The prognostic value of the CP score has been substantiated in numerous studies, further solidifying its importance in the management of patients with decompensated cirrhosis. Nevertheless, incorporation of two subjective variables (encephalopathy and ascites), along with potential interobserver variability, has driven the pursuit of more unbiased and precise prognostic markers in this context.

Indeed, creatinine serum levels have been demonstrated to serve as a dependable predictor of survival in individuals with cirrhosis both during the acute complication phase of the disease and over its natural course. In this work, we tried to see if serum creatinine levels might be added to the standard CP score to improve it.

## Materials and methods

This analytical cross-sectional study primarily aimed to contrast the predictive accuracy of the creatinine-modified Child-Pugh (CP) score with that of the traditional CP score and secondary endpoints were hospital duration of more than 14 days and mortality. The sensitivity, specificity, and receiver operating characteristics (ROC) curves were used to analyze the scores for hepatic encephalopathy. The study was approved by the Institutional Ethics Committee of Sarojini Naidu Medical College, Agra (#SNMC/IEC/Thesis/2023/175). Patients were enrolled sequentially upon their presentation to the medicine department. The research involved 100 decompensated cirrhosis patients admitted to the medicine department from November 2020 to April 2022. Patients were followed up till the hospital course. Exclusion criteria included hepatocellular carcinoma, intrinsic renal disease, and severe primary cardiopulmonary failure. For patients with multiple readmissions during the study period, their initial admission was the only one considered.

Diagnosis of decompensated cirrhosis required a combination of clinical cirrhosis signs, laboratory evidence of cirrhosis, radiographic cirrhosis findings as well as one or more indicators of liver decompensation, such as nonobstructive jaundice, variceal hemorrhage, ascites, or hepatic encephalopathy. All patients with decompensated cirrhosis received a thorough physical examination, comprehensive laboratory testing, and an extensive medical history upon admission. Laboratory tests included hemogram with cell counts, coagulation profile, and kidney and liver function tests. Patients are classified into three groups based on their CP score as follows: Class A (5-6), Class B (7-9), and Class C (10-15). The CP score, a comprehensive measure, takes into account serum bilirubin levels, albumin levels, prothrombin time prolongation, hepatic encephalopathy, and ascites severity. It ranges from 5 to 15.

The severity of hepatic encephalopathy is classified into five grades, ranging from Grade 0 (minimal hepatic encephalopathy {HE}, no clinical manifestations) to Grade IV (coma, unresponsiveness). The modified Child-Pugh score was computed by incorporating serum creatinine as a sixth variable. If the creatinine level was less than 1.3 mg/dL, the score was zero; if it was between 1.3 and 1.8 mg/dL, the score was two; and if it was more than 1.8 mg/dL, the score was four.

Statistical analysis

SPSS (Chicago, IL: IBM Corp.) was employed for statistical evaluation. Data was visually depicted using mean (standard deviation), frequency count, and proportion (%). Independent t-tests were implemented to contrast discrete variables between groups, whereas chi-square tests were utilized for categorical variables. The receiver operating characteristics (ROC) curve was leveraged to compare two scores. A p-value below 0.05 was deemed statistically significant.

## Results

The age distribution of the patients was as follows: 16.0% (n=16) were between 21 and 30 years, 29.0% (n=29) were between 31 and 40 years, 26.0% (n=26) were between 41 and 50 years, 18.0% (n=18) were between 51 and 60 years, and 11.0% (n=11) were over 60 years. The patients had a mean age of 44.71 years and a standard deviation of 13.40 years. Out of 100 patients, 74% (n=74) were male and 26%(n=26) were female, and among them, 55% (n=55) were alcoholics, while 45% (n=45) were non-alcoholics (Table 1).

**Table 1:** Basic characteristics of the patients. The data are represented as n, percentage, mean±SD. P<0.05 is considered significant.

Variables		n	Percentage
Age (years)	21-30 years	16	16.0%
	31-40 years	29	29.0%
	41-50 years	26	26.0%
	51-60 years	18	18.0%
	>60 years	11	11.0%
	Mean±SD	44.71±13.40, p-value=0.005	
Gender	Male	74	74.0%
	Female	26	26.0%
Addiction	Alcoholic	55	55.0%
	Non-alcoholic	45	45.0%

The number of cases of mild, moderate, and severe hepatic encephalopathy was 52, 24, and 24, respectively. The mean age was not significantly different between mild, moderate, and severe hepatic encephalopathy groups (Table 2). No patient presented in Child-Pugh Class A.

**Table 2:** Comparison of mean age between mild, moderate, and severe hepatic encephalopathy. The data are represented as n and mean±SD. P-value<0.05 is considered significant.

Variables	Mild (n=52)		Moderate (n=24)		Severe (n=24)		f	p-Value
	Mean	±SD	Mean	±SD	Mean	±SD		
Age (years)	43.81	13.48	46.63	12.62	44.75	14.34	0.36	0.700

Nearly all blood parameters didn’t differ significantly between mild, moderate, and severe hepatic encephalopathy. This was in contrast to mean serum urea and serum creatinine which was significantly higher in severe hepatic encephalopathy than in mild and moderate encephalopathy (Table 3).

**Table 3:** Comparison of mean biochemical parameters between mild, moderate, and severe hepatic encephalopathy. The data are presented as n and mean±SD. *P-value <0.05 is considered significant. TLC: total leucocyte count; ALT: alanine transaminase; AST: aspartate aminotransferase; ALP: alkaline phosphatase; ALB: albumin; PT: prothrombin time; INR: international normalized ratio

Variables	Mild (n=52)		Moderate (n=24)		Severe (n=24)		f	p-Value
	Mean	±SD	Mean	±SD	Mean	±SD		
HB (g/dL)	8.49	2.10	8.58	2.65	8.70	2.11	0.07	0.928
TLC x10^3 ^(cells/mm^3^)	9.27	5.99	9.732	6.30	9.57	0.56	0.05	0.947
Neutrophils (%)	73.83	10.58	73.75	11.18	72.83	15.23	0.06	0.942
Lymphocytes (%)	20.40	8.97	20.13	9.26	16.75	8.70	1.45	0.240
Platelet count ×10^5^ (cells/mm^3^)	1.05	0.68	1.05	0.75	0.90	0.65	0.47	0.624
Serum bilirubin (mg/dL)	4.37	5.27	4.12	5.77	8.21	9.81	3.10	0.050
Serum ALT (IU/L)	116.52	179.87	126.04	132.95	84.16	88.77	0.53	0.590
Serum AST (IU/L)	149.80	291.52	177.00	321.39	146.33	182.36	0.09	0.914
Serum ALP (IU/L)	217.04	128.80	282.75	294.20	300.63	323.51	1.26	0.289
Serum ALB (g/dL)	2.62	0.54	2.45	0.57	2.66	0.52	1.10	0.337
Serum urea (mg/dL)	39.95	22.35	54.54	26.14	90.28	53.79	18.30	<0.001^*^
Serum creatinine (mg/dL)	0.99	0.39	1.39	0.46	2.42	1.11	38.64	<0.001^*^
PT (seconds)	23.74	12.89	20.96	8.69	21.79	6.75	0.64	0.531
INR	2.10	1.16	1.59	0.92	1.80	0.64	2.27	0.108

In the patient population, ascites were the most prevalent manifestation, with refractory ascites constituting 11% (n=11) of the overall cases. This was followed by hematemesis which was present in 40% (n=40) cases. Regarding the outcomes, 30% (n=30) of patients succumbed to cirrhosis complications, and 60% (n=60) patients experienced hospital stays exceeding 14 days. Of the 30 deceased patients, six patients belonged to Child-Pugh Class B, while 24 belonged to Class C and no cases of Child-Pugh Class A were observed (Table 4).

**Table 4:** Clinical history and outcomes of patients with decompensated cirrhosis (n=100). The data are presented as n and percentage.

Qualitative variables	Frequency (n)	Percentage
History of ascites	100	100%
History of refractory ascites	11	11%
History of hematemesis: positive	40	40%
History of hematemesis: negative	60	60%
Outcomes: death	30	30%
Outcomes: hospital stay >14 days	60	60%
Mortality analysis (n=30): Class A	0	0%
Mortality analysis (n=30): Class B	6	20%
Mortality analysis (n=30): Class C	24	80%

The number of cases of mild, moderate, and severe hepatic encephalopathy was 52, 24, and 24, respectively. In the context of mild hepatic encephalopathy, 25% (n=13) of cases were classified as Child-Pugh Class B, while 75% (n=39) were Class C. For moderate encephalopathy, 4.17% (n=1) were Class B and 95.83% (n=23) were Class C. In severe encephalopathy, there were no cases in Class B and all cases were in Class C (Table 5). The incidence of Child-Pugh Class B significantly decreased in moderate and severe hepatic encephalopathy compared to mild encephalopathy. The incidence of Child-Pugh Class C significantly increased in moderate and severe encephalopathy.

**Table 5:** Comparison of frequencies of Child-Pugh Class A, Child-Pugh Class B, and Child-Pugh Class C in between mild, moderate, and severe hepatic encephalopathy. The data are presented as n and percentage. *P-value <0.05 is considered significant.

Variables	Mild (n=52)		Moderate (n=24)		Severe (n=24)		f	p-Value
	n	%	n	%	n	%		
Child-Pugh Class A	0	0.00	0	0.00	0	0.00	8.50	0.014^*^
Child-Pugh Class B	13	25.00	1	4.17	0	0.00		
Child-Pugh Class C	39	75.00	23	95.83	24	100		

**Figure 1:**
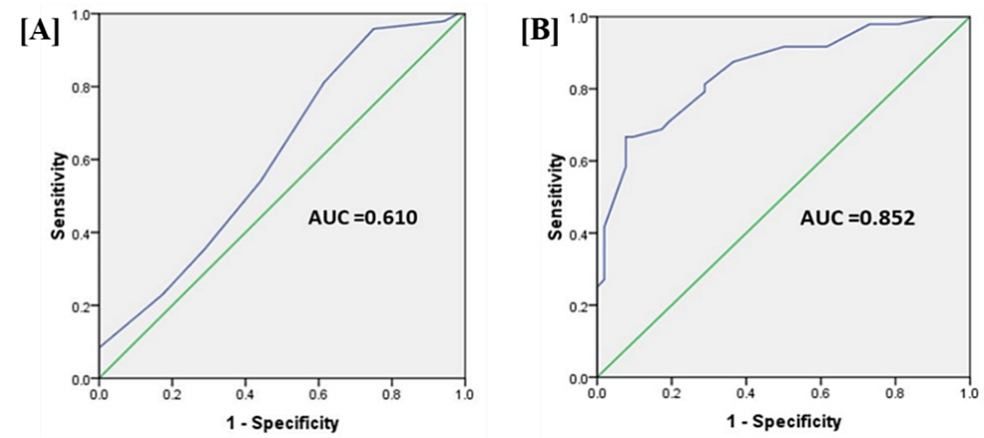
ROC curve analysis of moderate and severe hepatic encephalopathy patients. The images show (A) Child-Pugh and (B) creatinine-modified Pugh scores. Each receiver characteristic curve is expressed as a solid line. The data are presented as sensitivity and specificity and ROC analysis as AUC. ROC: receiver operating characteristic; AUC: area under the curve

The Child-Pugh score did not exhibit a significant area under the curve (AUC) for moderate and severe hepatic encephalopathy on the ROC curve (AUC=0.610). In contrast, the creatinine-modified Child-Pugh score for moderate and severe hepatic encephalopathy demonstrated a significantly large AUC on the ROC curve (AUC=0.852) (Table 6 and Figures 1A, 1B).

**Table 6:** Sensitivity and specificity and receiver operating characteristic (ROC) analysis of Child-Pugh for moderate and severe hepatic encephalopathy. The data are presented as sensitivity and specificity and ROC analysis as area under the curve. *P-value <0.05 is considered significant.

Variables	Cut-off	Sensitivity	Specificity	Area	95% CI		p-Value
					Lower	Upper	
Child-Pugh score	10	81.2%	38.5%	0.610	0.499	0.720	0.059
Creatinine-modified Child-Pugh score	1.20	79.2%	71.8%	0.852	0.777	0.926	<0.001^*^

## Discussion

The CP score is usually employed in routine practice for evaluating the prognosis in liver disease patients, owing to their straightforward application in everyday clinical settings [9]. While prior research has shown the predictive importance of the CP score, its reliance on two subjective variables - encephalopathy and ascites, introduces interobserver variability, prompting research into more unbiased and precise prognostic markers. Our research objectives were to examine the in-hospital results of a patient group with cirrhosis and contrast the prognostic power of the CP score with the creatinine-modified CP score.

The majority of patients in our study (55%, n=55) fell between the ages of 30 and 50. The average age of the cirrhotic patients in our study was 44.71±13.40 years (Table 1). Liver cirrhosis was found to be more prevalent in males in our study (74%, n=74), consistent with prior research done in Pakistan and Indonesia [10,11]. Of the 100 patients in our study, 52% (n=52) experienced mild hepatic encephalopathy, 24% (n=24) experienced moderate hepatic encephalopathy, and 24% (n=24) experienced severe hepatic encephalopathy (Table 2). Previous studies have implicated that blood creatinine levels serve as a significant predictor of mortality in cirrhotic patients, regardless of the presence of liver disease complications [12-14]. This was observed in our study, where moderate to severe hepatic encephalopathy was associated with significantly higher serum creatinine levels (p<0.001) (Table 3). 

Child-Pugh Class A was not encountered in this study. For mild hepatic encephalopathy, Child-Pugh Class B and C constituted 25.00% (n=13) and 75.00% (n=39), respectively; for moderate hepatic encephalopathy, they were 4.17% (n=1) and 95.83% (n=23), and for severe hepatic encephalopathy, 0% and 100% (Table 5). The occurrences of Child-Pugh Class B were substantially lower in moderate and severe hepatic encephalopathy compared to mild hepatic encephalopathy, while the occurrences of Child-Pugh Class C were substantially higher. A previous study done in Greece found that the modified Child-Pugh score, which included serum creatinine as a trichotomous categorical variable and assigned points for each value (0 points for creatinine 1.3 mg/dL, 2 points for creatinine=1.3-1.8 mg/dL, and 4 points for creatinine >1.8 mg/dL), was better than Child-Pugh score in predicting the prognosis in decompensated cirrhosis patients [15]. This is consistent with our findings of a substantially large area under the curve (AUC=0.852) on the ROC for moderate and severe hepatic encephalopathy when utilizing the modified CP score, with specificity and sensitivity of 71.8% and 79.2%, respectively. This observation contrasts with the CP score, which did not exhibit a significantly large area under the curve (AUC=0.610) on the ROC for moderate and severe hepatic encephalopathy (Table 6 and Figures 1A, 1B).

According to a systematic review conducted in Italy on approximately 118 publications, the one-year mortality rate for decompensated cirrhosis ranges from 20% to 57% [16]. Similar to this, the current study found that 30% (n=30) of patients succumbed to cirrhosis complications during the initial hospital stay. Among the 30 deceased patients, six patients belonged to Child-Pugh Class B, while 24 belonged to Class C. To lower the death rate, it’s imperative to recognize those who might have a poor prognosis and admit them to the liver ICU or refer them to transplantation.

Due to their simplicity and enhanced predictive power over the conventional CP score, creatinine-modified CP scores seem worthy of further investigation. While the Model for End-Stage Liver Disease (MELD) score provides a more accurate assessment of liver disease severity, its complexity necessitates the use of electronic devices and prevents its immediate application at the bedside. Recognizing the limitations of Child-Pugh scores, the authors recognize a distinct advantage of the creatinine-modified CP score over the original CP score in predicting survival among liver cirrhosis patients. For a number of reasons, the creatinine-modified CP score was developed. As our study demonstrated, blood creatinine levels are a significant and reliable indicator of death in individuals with liver cirrhosis, regardless of whether they also have liver disease complications. 

Study limitations

First, for patients with multiple readmissions during the study period, their initial admission was the only one considered. Second, CP and modified CP scores were assessed based on the data and the clinical profile at the time of admission only. The hospital course was not taken into account when classifying the patients into Child-Pugh classes. Third, cirrhosis-related skeletal muscle atrophy results in sarcopenia which itself is associated with higher rates of liver cirrhosis complications. It along with impaired hepatic creatine production impacts serum creatinine levels. This might have resulted in a low mean serum creatinine calculation.

## Conclusions

We found that the original Child-Pugh score was not as good at predicting encephalopathy as the creatinine modified CP score, and this was especially true when moderate and severe encephalopathy was present. Additionally, we found that patients with moderate and severe hepatic encephalopathy had mean serum creatinine levels that were considerably high. This further implicates serum creatinine as the sixth component in the CP score. Our findings lead us to suggest that in regular clinical practice, the CP score need to be substituted with the creatinine modified CP score. Extensive research is imperative to delineate the clinical utility of serum creatinine in liver cirrhosis.
